# A public dataset of overground and treadmill walking kinematics and kinetics in healthy individuals

**DOI:** 10.7717/peerj.4640

**Published:** 2018-04-24

**Authors:** Claudiane A. Fukuchi, Reginaldo K. Fukuchi, Marcos Duarte

**Affiliations:** 1 Neuroscience and Cognition Program, Federal University of ABC, Sao Bernardo do Campo, Brazil; 2 Biomedical Engineering Program, Federal University of ABC, Sao Bernardo do Campo, Brazil

**Keywords:** Gait, Biomechanics, Kinematics, Kinetics, Human movement

## Abstract

In a typical clinical gait analysis, the gait patterns of pathological individuals are commonly compared with the typically faster, comfortable pace of healthy subjects. However, due to potential bias related to gait speed, this comparison may not be valid. Publicly available gait datasets have failed to address this issue. Therefore, the goal of this study was to present a publicly available dataset of 42 healthy volunteers (24 young adults and 18 older adults) who walked both overground and on a treadmill at a range of gait speeds. Their lower-extremity and pelvis kinematics were measured using a three-dimensional (3D) motion-capture system. The external forces during both overground and treadmill walking were collected using force plates and an instrumented treadmill, respectively. The results include both raw and processed kinematic and kinetic data in different file formats: c3d and ASCII files. In addition, a metadata file is provided that contain demographic and anthropometric data and data related to each file in the dataset. All data are available at Figshare (DOI: 10.6084/m9.figshare.5722711). We foresee several applications of this public dataset, including to examine the influences of speed, age, and environment (overground vs. treadmill) on gait biomechanics, to meet educational needs, and, with the inclusion of additional participants, to use as a normative dataset.

## Introduction

Gait analysis (GA) has been widely used to better understand the gait patterns of a wide range of populations. The application of this method has the ability to distinguish between normal and abnormal gaits (Gage et al., 2009), to determine the best intervention (Kay et al., 2000; Lofterod et al., 2007; Wren et al., 2011), and to detect pathologies at subclinical stages (Carpinella et al., 2007; Rao et al., 2008). These measures are objective and are typically performed using a three-dimensional (3D) motion-capture system and force plates.

A typical clinical study commonly approaches GA by comparing a group of pathological (e.g., post-stroke) individuals with able-bodied controls. However, the control group usually consists of a small number of age-matched individuals, each walking at a comfortable speed, which is commonly faster than that of individuals in the pathological group (Marrocco et al., 2016). Therefore, the validity of these studies is limited by the potential bias caused by the difference in gait speeds. A possible solution to this problem is to perform walking trials at a wider range of gait speeds, from very slow to very fast, to enable comparisons that are less biased. Previous studies have reported speed dependency in kinematics and kinetics data during overground walking (Bovi et al., 2011; Schwartz, Rozumalski & Trost, 2008). However, the authors of these studies provided only the average (and standard deviation) data across participants, and no raw data were publicly available with which to validate the inferences made by the studies. In fact, recently, data sharing and increased acceptance of replication studies have been advocated to overcome the aforementioned limitations and to validate the inferences made by previous gait studies (Ferber et al., 2016; Knudson, 2017). Unfortunately, so far, only a handful of walking biomechanics datasets have been made publicly available (Hnat, Moore & Van den Bogert, 2015; Hodgins, 2015; Kirtley, 2014; Moore, Hnat & Van den Bogert, 2014; Willson & Kernozek, 2014).

Furthermore, other studies have advocated the need to share data and the importance of a normative database (Winter, 1993) to improve the interpretation of GA outcomes. In the early 1990s, Winter began to make gait datasets available in his book (Winter, 2009); however, the only data provided were those of a single healthy subject. A few other gait normative datasets are available in the literature (Fukuchi, Fukuchi & Duarte, 2017; Moore, Hnat & Van den Bogert, 2015; Van den Bogert et al., 2013; Wang & Srinivasan, 2014), and although these datasets are valuable for a wide range of applications, their usefulness is lessened because they are usually limited to a single type of data (e.g., kinematics data), one walking surface (either overground or treadmill), and one gait speed (e.g., a self-selected speed).

To address these limitations, this study aimed to create a publicly available dataset of 3D walking kinematics and kinetics data on healthy young and older adults at a range of gait speeds in both the treadmill and overground environments.

## Methods

To generate data for the dataset, we measured the kinematics and kinetics of participants walking at various speeds both overground and on a treadmill.

### Participants

Study participants included 42 volunteers, including 24 young adults (age 27.6 ± 4.4 years, height 171.1 ± 10.5 cm, and mass 68.4 ± 12.2 kg) and 18 older adults (age 62.7 ± 8.0 years, height 161.8 ± 9.5 cm, and mass 66.9 ± 10.1 kg). All participants were free of any lower-extremity injury in the last six months before the data were collected, and all were free of any orthopedic or neurologic disease that could interfere with their gait patterns. In order to train with the equipment and design appropriate procedures, a pilot study was conducted first with five participants. The provided metadata file, WBDSinfo.xlsx, contains the demographic and anthropometric data of the participants. Prior to the collection of data, each participant read and signed a consent form that had previously been approved by the university ethics committee (CAAE: 53063315.7.0000.5594).

### Data acquisition

Standard gait-analysis procedures were employed to collect data using a motion-capture system that had 12 cameras (Raptor-4; Motion Analysis Corporation, Santa Rosa, CA, USA), five force platforms (three 40 × 60-cm Optima models; AMTI, Watertown, MA, USA; two 40 × 60-cm 9281EA models; Kistler, Winterthur, Switzerland) embedded in the floor, and a dual-belt, instrumented treadmill (FIT; Bertec, Columbus, OH, USA) in a 10 × 12-m room at the Laboratory of Biomechanics and Motor Control at the Federal University of ABC, Brazil. The instrumented treadmill has handrails alongside it attached directly to split mounting plates. Therefore, while the subjects may hold the handrails during gait, the measured forces include only the forces applied by the legs during stance. The kinematic data were acquired at 150 Hz, and the data on ground-reaction forces were acquired at 300 Hz using a motion-capture system (Cortex 6.0; Motion Analysis, Santa Rosa, CA, USA).

### Procedures

All gait trials were performed in barefoot conditions, and the participants wore comfortable shorts (women also wore sports bras). Each participant was asked to perform overground walking trials, first at a self-selected comfortable speed, and then at speeds 30% faster and 30% slower than the comfortable speed. In addition, the participants walked on the treadmill at eight different controlled speeds, which are described below. Previously, a computerized random-number generator had been used to define the order of the walking trials on the treadmill. The marker-set protocol adopted for this study comprised 26 anatomical reflective markers (Leardini et al., 2007), and additional markers were used on the iliac crests to enable future data users to define alternative anatomical and technical coordinate systems for the pelvis (Kisho Fukuchi et al., 2010) (see Table 1 in the Supplementary material). The following data-collection procedures were implemented.

**Figure 1:**
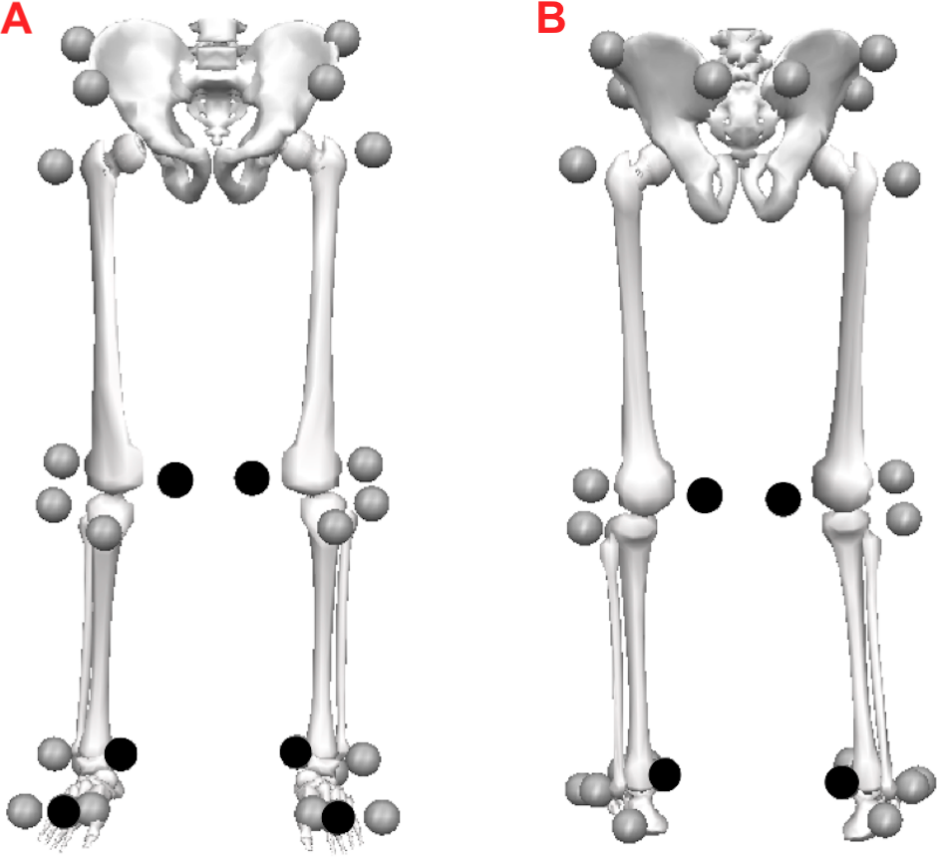
Marker-set protocol. Location of reflective markers for the pelvis segment and lower extremities during the static condition in the anterior (A) and posterior (B) views. During the walking trials, the markers shown in black were removed.

1. Prior to data collection, each participant received a brief explanation of the study and signed the consent form.
2. Body height and body mass were measured.
3. Leg length was measured by assessing the distance from the anterior superior iliac spine (ASIS) to the ipsilateral medial malleolus while the participant lay in a supine position.
4. Markers were placed directly onto the skin in the pelvic and lower-extremity segments (Leardini et al., 2007) (Fig. 1).
5. A standing anatomical-calibration trial was performed with the participant standing still for 1 s with the arms crossed in front of the trunk and the feet in a standard position parallel to the *X*-axis of the laboratory coordinate system (LCS) (Fukuchi, Fukuchi & Duarte, 2017). A template was used to ensure that the long axes of the feet were aligned with the *X*-axis of the LCS.
6. After the calibration trial, the medial epicondyle, medial malleolus, and second metatarsal head markers were removed from the right and left foot.
7. To determine each participant’s comfortable speed, after a familiarization period, gait speed was measured during three walking trials along a 10-m walkway from start and end at rest, at the participant’s self-selected comfortable speed. The average speed from across these trials was deemed the comfortable speed.
8. To determine the dimensionless gait speed, the Froude number, *v*^∗^, was calculated based on the participant’s average self-selected comfortable speed, *v*, and leg length, *l*_0_, (Hof, 1996):
  \begin{eqnarray*}& & {v}^{\ast }=v/\sqrt{g{l}_{0}}, \text{where} g \text{is} 9.81 {\text{m/s}}^{\text{2}}. \end{eqnarray*}
9. Participants first performed overground walking trials at their self-selected comfortable speed, and then at speeds 30% faster and 30% slower than their comfortable speed.
10. After this, they were asked to walk on the treadmill, and the following protocol was performed.
  a. To familiarize themselves with the treadmill speed, participants walked at their comfortable speed for 5 min.
  b. Then, each participant walked for 90 s at each of the eight gait-speed conditions (40%, 55%, 70%, 85%, 100%, 115%, 130%, and 145% of the self-selected dimensionless speed (Froude number)) in a randomized order. At each speed, the kinematic and kinetic data were recorded for the last 30 s of the trial. During the gait trials, the participants were asked to walk naturally and were allowed to hold the handrails of the treadmill if necessary.
11. After the treadmill task, each participant’s overall perceived exertion was measured using the Borg (6–20) Perceived Exertion Scale (Utter et al., 2004).

### Data processing

The data processing was performed using Cortex software version 6.0 (Motion Analysis, Santa Rosa, CA, USA) using procedures similar to those previously reported by Fukuchi, Fukuchi & Duarte (2017).

Visual 3D software version 6.00.33 (C-motion Inc., Germantown, MD, USA) was used to perform all kinematics and kinetics calculations. To enable users to process the data in the Visual 3D software, a Visual 3D pipeline file, WBDSpipelineV3D.v3s, is available at Figshare (DOI: 10.6084/m9.figshare.5722711). In addition, a metadata file in .xlsx format, WBDSinfo, contains the data related to the treadmill and overground files. The analysis of the overground trials considered only those files that contained at least one full gait cycle (stance and swing phase) detected using force plates. However, further trials (i.e., incomplete gait cycles) are provided so that prospective users can decide what data to consider in their own analyses. In all, 1,409 trials (right side: 657; left side: 752) contained a full gait cycle, and 1,233 trials (right side: 685; left side: 548) contained only the stance phase of the gait. The public dataset consists of raw c3d files and ASCII files containing both 3D marker coordinates and external forces. In addition, time-normalized kinematic and kinetic average curves, which were considered processed data, were calculated for each participant for each walking condition tested (overground and treadmill at various gait speeds).

## Results

### Raw data

The files containing the raw data are available at Figshare (DOI: 10.6084/m9.figshare.5722711) in both c3d format and ASCII file format. The c3d files can store both the 3D coordinates of the markers (mkr) and the force signals (grf) in the same file. Separate text files were generated for the markers and force signals, as the sample frequencies of the kinematics and kinetics data differed. In addition, the data related to the static trial (the standing anatomical-calibration trial), which contain only marker trajectories, are available in both the c3d and the text formats. The metadata file, WBDSinfo.xlsx, provides a full description of these files. Furthermore, the text files also contain the time-normalized ensemble average of the kinematics and kinetics curves for each participant at each gait speed and for each environment condition (overground and treadmill). The total number of gait trials is not the same across participants because it reflects the variation in the number of valid trials per participant.

The files provided are labeled “WBDS”, which stands for Walking Biomechanics Dataset; “xx”, for the participant’s assigned number (from 01 to 42); and “walk”, for the walking task. After this labeling, the following specific notations are used.

• Environment: “O” for overground and “T” for treadmill.
• Trial: “yy” indicates the trial number assignment for the overground condition only.
• Speed: “01” to “08”, which corresponds to the treadmill trials at 40%, 55%, 70%, 85%, 100%, 115%, 130%, and 145% of the self-selected, dimensionless speed (Froude number); and “S”, “C”, or “F”, which correspond to the slow, comfortable, and fast speeds for the overground trials.

For example, a file named “WBDS01walkO01Smkr.txt” indicates that the file contains the marker-coordinate (mkr) data of the first participant performing the first overground trial at the slowest speed. Similarly, “WBDS01walkT01mkr.txt” indicates that the file contains the marker-coordinate (mkr) data of the first participant walking at the treadmill speed corresponding to 40% of the self-selected dimensionless speed. The c3d files contain the 3D coordinates of the 28 markers in the static trial (for example, WBDS01static1.c3d) and the coordinates of the 22 markers and the force data during the walking trials (for example, WBDS01walkT01.c3d). The force data during the walking trials were also provided as plain-text files consisting of a time column (nth frame number), the forces (*Fx*, *Fy*, and *Fz* in Newtons), the center of pressure (COPx, COPy, and COPz in mm), and the free moment about the vertical axis (Ty in Nm). The force-data files regarding the overground condition contain 36 columns corresponding to the time column along with the data from the five force plates. In contrast, the force-data files regarding the treadmill condition contain 15 columns corresponding to the time column along with the data from the two force plates (left and right belt). An example of a MATLAB code demonstrates on how to read the data from these files and on how to conduct an exploratory data analysis.

### Metadata

A metadata file named WBDSinfo.xlsx is available at Figshare and contains the following data in various columns (the bold word in each of the following items corresponds to the heading of a column).

1. **Subject:** the index of the subject (from 01 to 42).
2. **FileName:** the filename of the walking trial (WBDSxx, where xx identifies the participant), including the format extensions (*.c3d or *.txt).
3. **AgeGroup:** the “Young” or “Older” group.
4. **Age:** the participant’s age in years.
5. **Height:** the participant’s height in centimeters, measured with a calibrated stadiometer.
6. **Mass:** the participant’s body mass in kilograms, measured with a calibrated scale.
7. **Gender:** the participant’s gender (M or F).
8. **Dominance:** preferred leg for kicking a ball (R or L).
9. **LegLength:** leg length in centimeters (the average of the two legs).
10. **Static1:** whether the corresponding walking trial was assigned (Yes or No) to the Static1 file.
11. **Static2:** whether the corresponding walking trial was assigned (Yes or No) to the Static2 file. The Static2 was performed due to technical issues (e.g., markers dropping off during the session, markers needing to be repositioned, etc.).
12. **GaitSpeed:** the walking velocity at each trial (m/s).
13. **TreadHands:** whether the participant walked while hanging onto the treadmill handrails during the whole walking trial (Yes) or not at all (No).
14. **FP_RightFoot:** the force-plate number that the participant hit with the right foot.
15. **FP_LeftFoot:** the force-plate number that the participant hit with the left foot.
16. **Notes:** text strings with any notes about the treadmill or the overground trials (“–” if the trial has no notes). Ex: “FP3 signal presented offset”.
17. **BorgScale:** the corresponding Borg Scale value.

In total, in both the c3d and txt formats, the WBDSinfo.xlsx file has 17 columns and 6,916 rows, corresponding to the total number of trials; the rows represent the static trial (*static1), the eight trials on the treadmill (*walkT01–T08), and the overground trials at the slow (*S), comfortable (*C), and fast (*F) speeds. The processed files of kinematics (*ang.txt) and kinetic (*knt.txt) data are also included. The number of rows varies for each participant, depending on the number of overground trials.

### Processed data

The ASCII files provide the ensemble average data for each participant throughout the full gait cycle (101 time-normalized points), which correspond to the time-normalized angles (pelvis, hip, knee, ankle, and foot), joint moments (hip, knee, and ankle), and GRF forces in the *X*, *Y*, and *Z* directions.

### Data exploration

The following is a partial exploratory analysis of the data. A companion MATLAB code provides examples of how these data can be explored. The curves shown in this section represent the ensemble average across all participants at a particular gait speed. The participants in the Young group (age range: 21–37 years) walked at eight speeds on the treadmill, whereas in the Older group (age range: 50–84 years), only 12 participants were able to walk at these eight speeds. To clarify: this section shows only the right leg and the pelvis curves of the Young group, both when walking overground and on the treadmill. The time-series curves of the Older group are shown in the Supplementary material.

#### Joint kinematics

**Figure 2:**
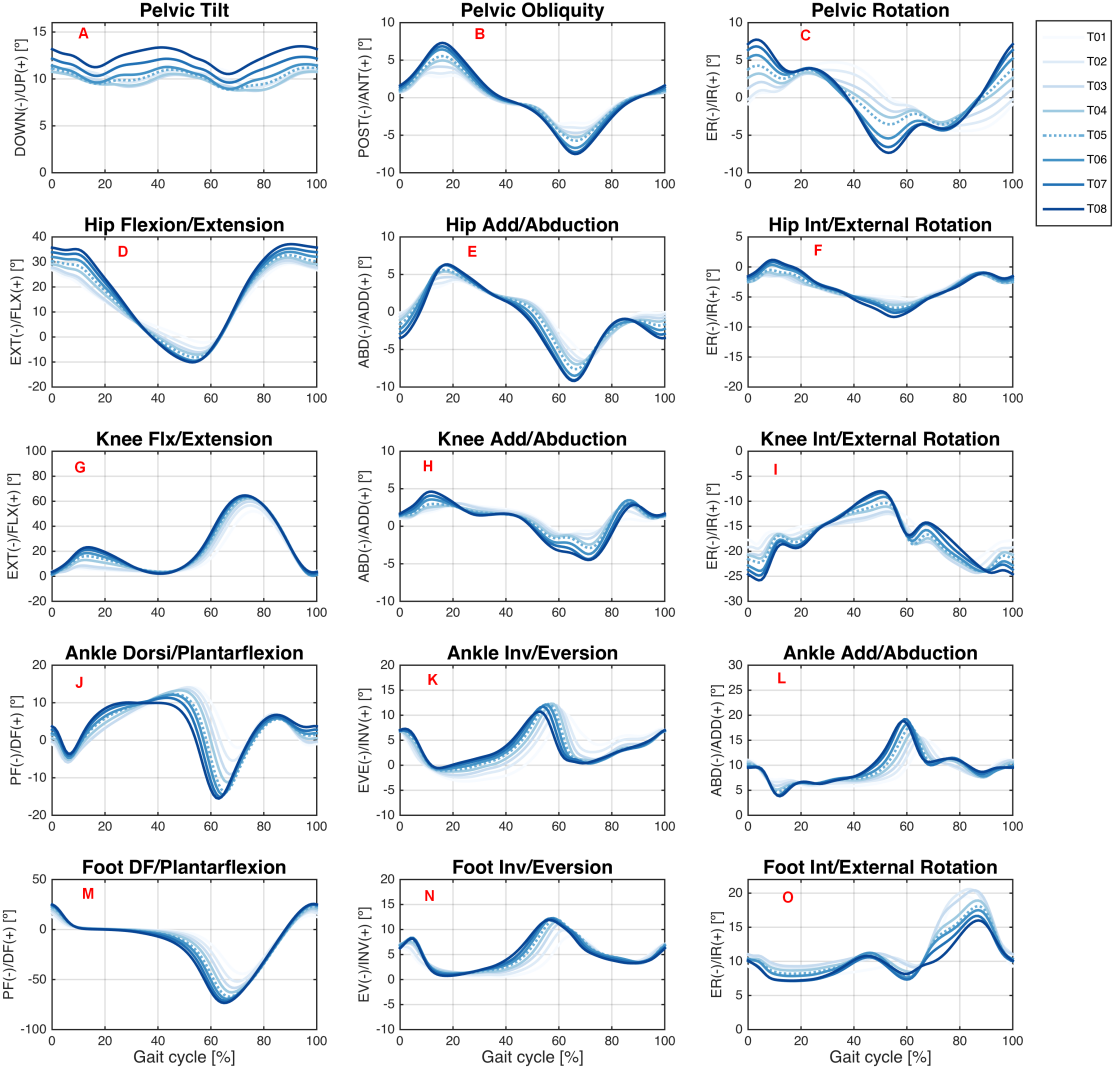
Angular kinematics during treadmill walking. Ensemble averages across Young group participants of the pelvic tilt (A), pelvic obliquity (B), pelvic rotation (C), hip flexion/extension (D), hip add/abduction (E), hip int/external rotation (F), knee flx/extension (G), knee add/abduction (H), knee int/external rotation (I), ankle dorsi/plantarflexion (J), ankle inv/eversion (K), ankle add/abduction (L), foot DF/plantarflexion (M), foot inv/eversion (N), and foot int/external rotation (O) angles during the treadmill walking condition. Each waveform represents a walking speed (light blue = T01, through dark blue = T08). The comfortable speed (T05) is represented by the dashed line.

**Figure 3:**
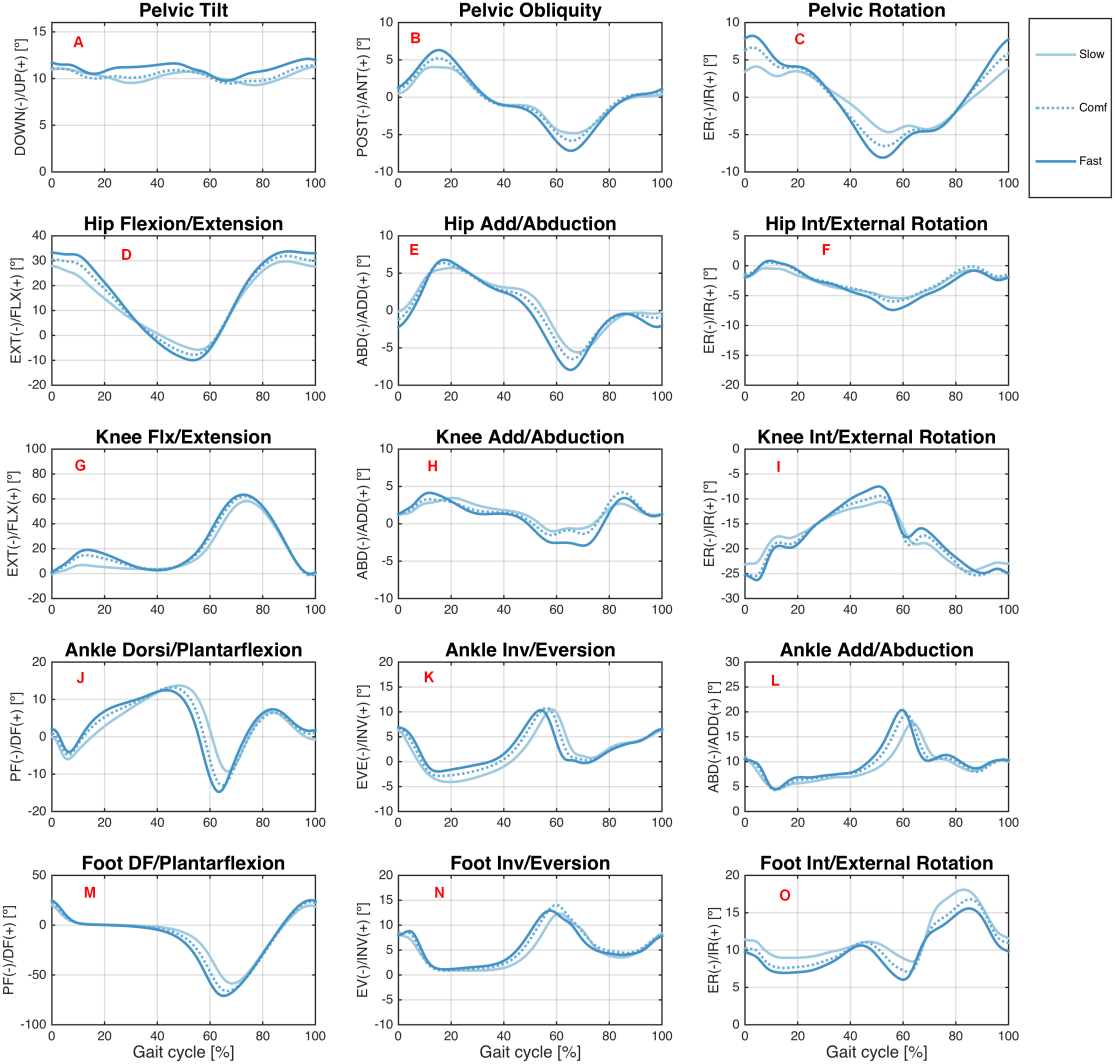
Angular kinematics during overground walking. Ensemble averages across Young group participants of the pelvic tilt (A), pelvic obliquity (B), pelvic rotation (C), hip flexion/extension (D), hip add/abduction (E), hip int/external rotation (F), knee flx/extension (G), knee add/abduction (H), knee int/external rotation (I), ankle dorsi/plantarflexion (J), ankle inv/eversion (K), ankle add/abduction (L), foot DF/plantarflexion (M), foot inv/eversion (N), and foot int/external rotation (O) angles during the overground walking condition. Each waveform represents a walking speed (light blue = slow, through dark blue = fast). The comfortable speed (Comf) is represented by the dashed line.

Figures 2 and 3 show the joint angles of the hip, knee, and ankle joints and of the pelvis and foot segments at the sagittal, frontal, and transverse planes, respectively, during treadmill and overground walking at various speeds.

#### Joint kinetics

**Figure 4:**
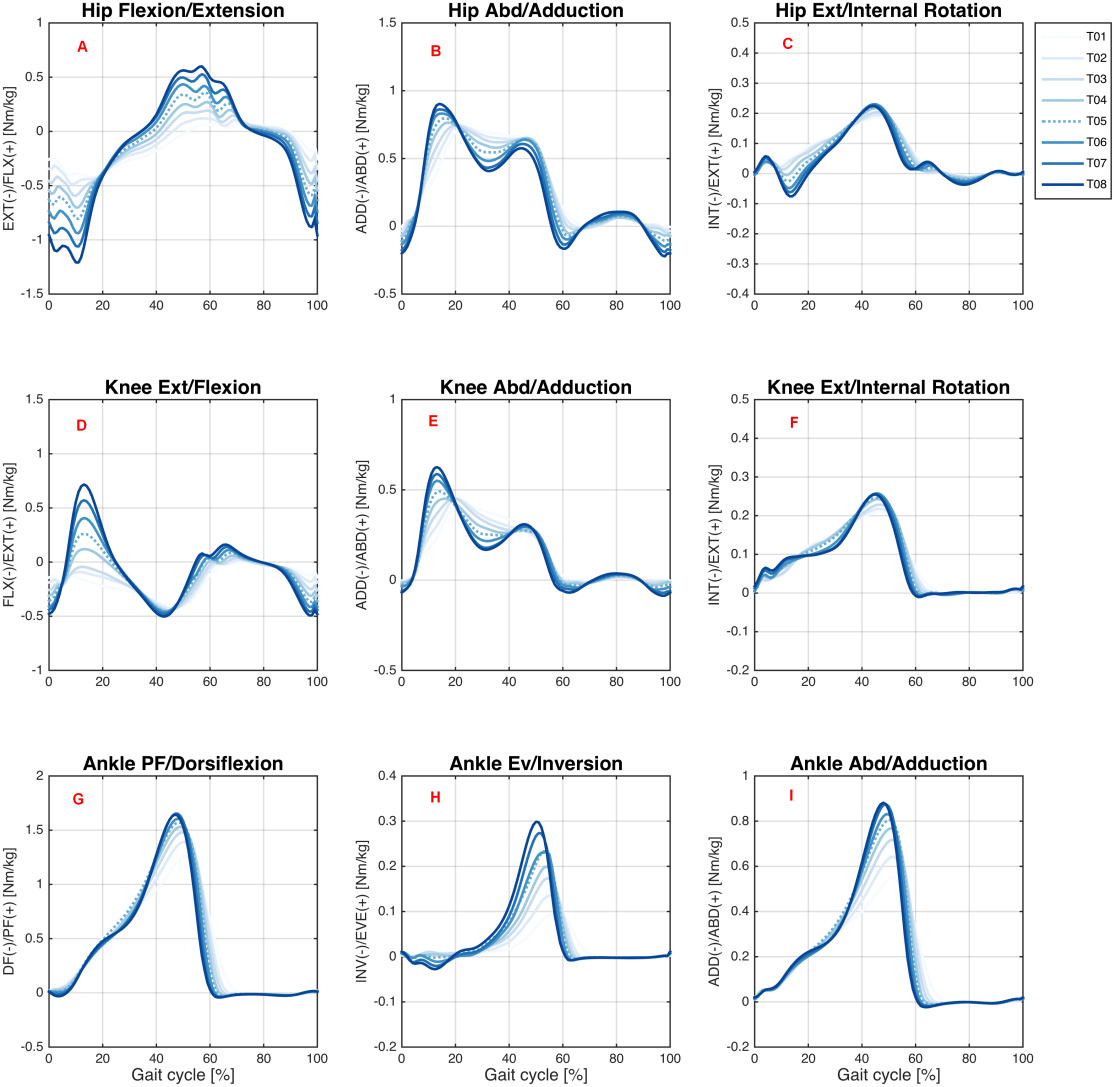
Joint moments during treadmill walking. Ensemble averages across Young group participants of the hip flexion/extension (A), hip abd/adduction (B), hip ext/internal rotation (C), knee ext/flexion (D), knee abd/adduction (E), knee ext/internal rotation (F), ankle PF/dorsiflexion (G), ankle ev/inversion (H), and ankle abd/adduction (I) joint moments during the treadmill walking condition. Each waveform represents a walking speed (light blue = T01, through dark blue = T08). The comfortable speed (T05) is represented by the dashed line.

**Figure 5:**
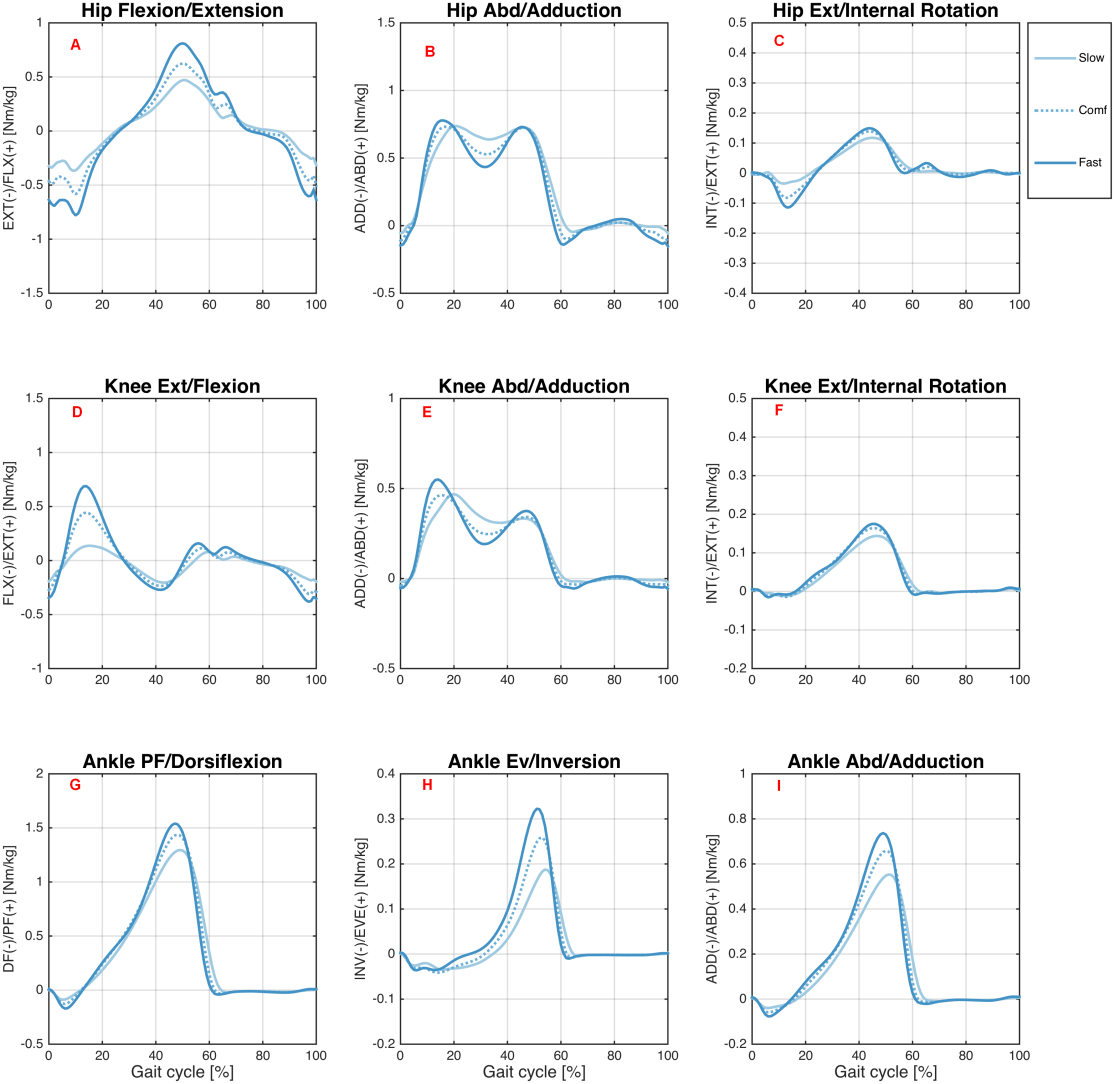
Joint moments during overground walking. Ensemble averages across Young group participants of the hip flexion/extension (A), hip abd/adduction (B), hip ext/internal rotation (C), knee ext/flexion (D), knee abd/adduction (E), knee ext/internal rotation (F), ankle PF/dorsiflexion (G), ankle ev/inversion (H), and ankle abd/adduction (I) joint moments during the overground walking condition. Each waveform represents a walking speed (light blue = slow, through dark blue = fast). The comfortable speed (Comf) is represented by the dashed line.

Figures 4 and 5 show joint moments for the hip, knee, and ankle joints during treadmill and overground walking, respectively, at various speeds.

#### Ground reaction forces (GRF)

**Figure 6:**
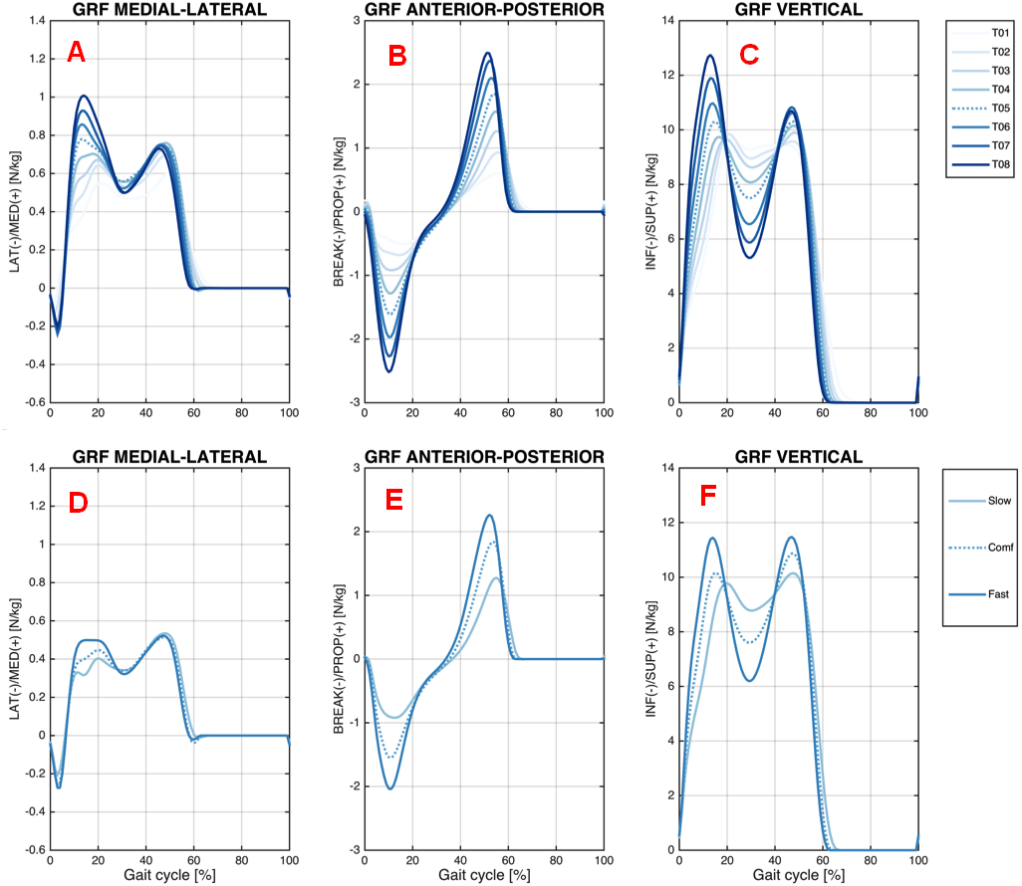
Ground reaction forces. Ensemble averages across Young group participants of the ground reaction force (GRF) on the treadmill (GRF medial-lateral (A), GRF anterior-posterior (B), and GRF vertical (C)); and overground (GRF medial-lateral (D), GRF anterior-posterior (E), and GRF vertical (F)) walking conditions. Each waveform represents a walking speed on the treadmill (light blue = T01, through dark blue = T08) and overground (light blue = Slow, through dark blue = Fast). The comfortable speed (T05 and Comf) is represented by the dashed line.

Figure 6 shows GRF data for the medial-lateral, anterior-posterior, and vertical direction during the treadmill and overground walking conditions at various speeds.

#### Young vs. older group

**Figure 7:**
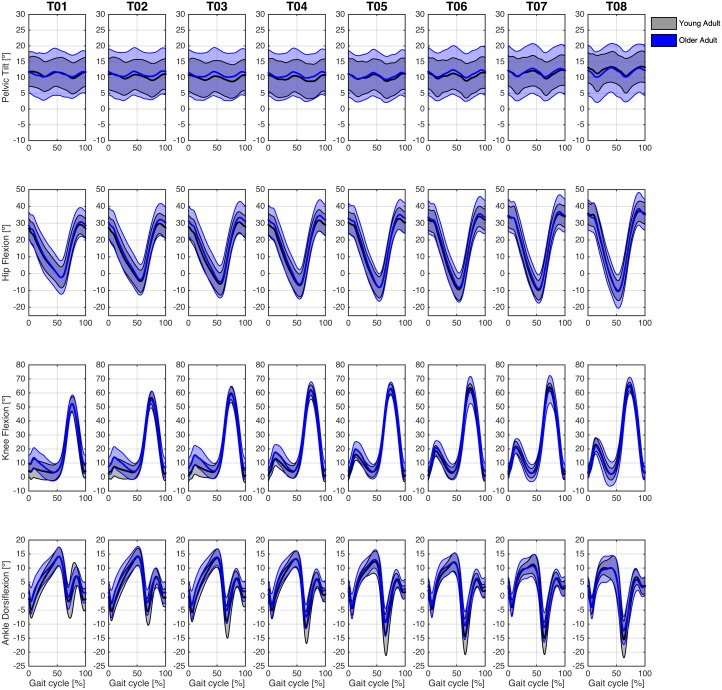
Angular kinematics during treadmill walking. Ensemble average ± 1 standard-deviation across participants of the pelvis, hip, knee, and ankle angles for the Young (grey curves) and Older (blue curves) groups at eight different gait speeds in the treadmill condition.

**Figure 8:**
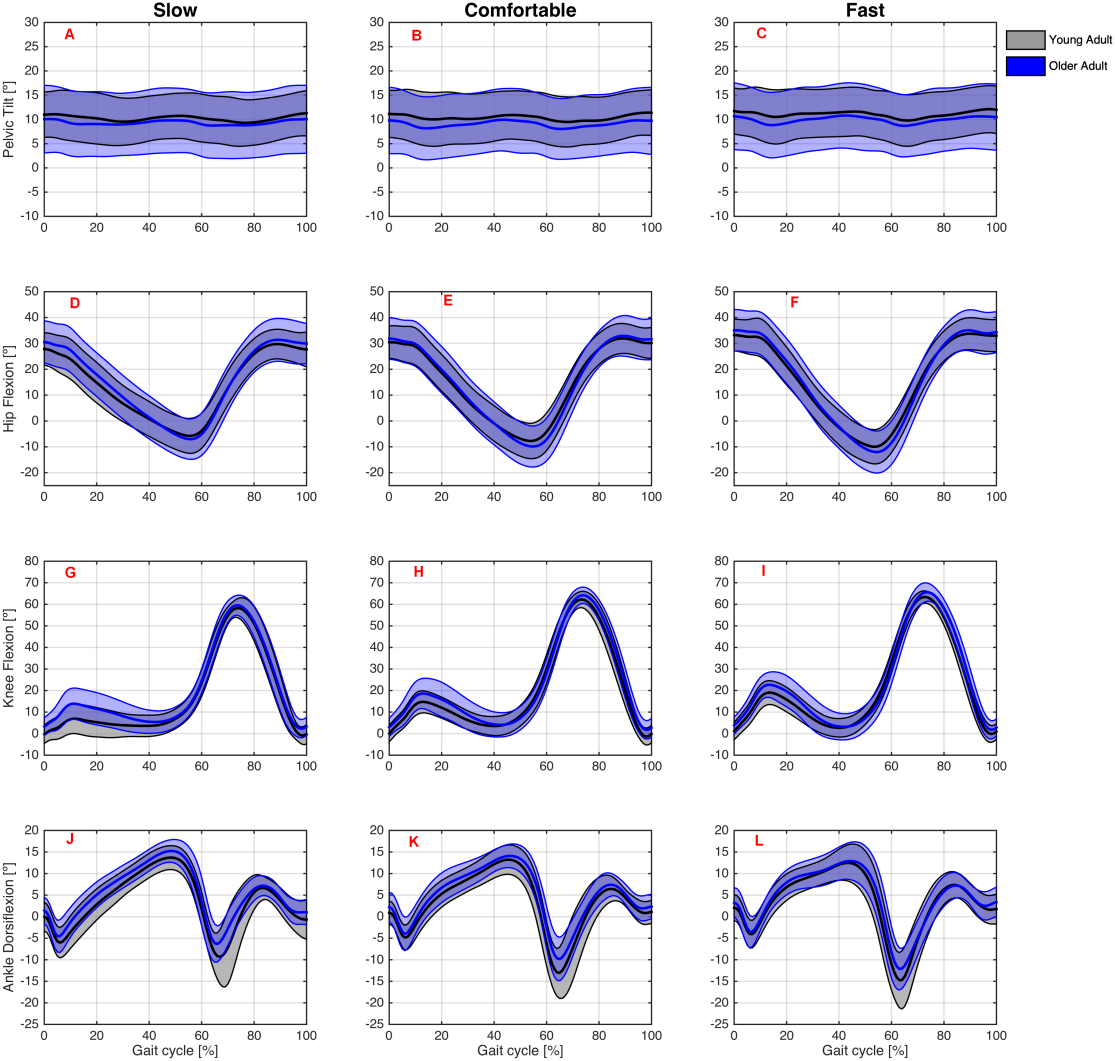
Angular kinematics during overground walking. Ensemble average ± 1 standard-deviation across participants of the pelvic tilt at the slow (A), comfortable (B), and fast (C) speeds; hip flexion at the slow (D), comfortable (E), and fast (F) speeds; knee flexion at the slow (G), comfortable (H), and fast (I) speeds; and ankle dorsiflexion at the slow (J), comfortable (K), and fast (L) speeds angles for the Young (grey curves) and Older (blue curves) groups during the overground walking condition.

We also present an exploratory analysis examining the kinematics patterns at the sagittal plane of the Young and Older groups at each treadmill walking speed (Fig. 7) and overground walking speed (Fig. 8).

## Discussion

This study presents a dataset of treadmill and overground walking kinematics and kinetics in a range of gait speeds for 24 healthy young individuals and 18 healthy older individuals. The study also makes available raw data comprising marker trajectories and GRFs and processed data comprising joint angles and joint moment waveforms that characterize the gait pattern of each participant. In addition, it makes available a file with metadata containing demographic data and file-related data, among other relevant data, and general notes pertaining to the experimental conditions.

Previous walking datasets with kinematics and kinetics data have been published elsewhere (Moore, Hnat & Van den Bogert, 2015; Van den Bogert et al., 2013). Moore, Hnat & Van den Bogert (2015) presented the gait data of 15 healthy adults walking at 3 different speeds, and Van den Bogert et al. (2013) presented the gait data of 12 healthy adults walking at comfortable speeds. Although these studies presented valuable information, the data provided referred only to young adults walking only on a treadmill. To our knowledge, the present study is the first to publicly provide a unique set of data that includes both young and older individuals walking in both overground and treadmill environments at a range of gait speeds. We foresee that the present dataset will add to the knowledge provided by previous studies that have examined gait changes related to the walking environment (e.g., overground vs. treadmill) (Alton et al., 1998; Parvataneni et al., 2009; Riley et al., 2007), age-related gait changes (Arnold et al., 2014; DeVita & Hortobagyi, 2000; Muir, Rietdyk & Haddad, 2014), and gait-speed changes (Chung & Wang, 2010; Hebenstreit et al., 2015; Kang & Dingwell, 2008) by enabling other groups to further address these issues in gait research, by, for example, applying various data-analysis techniques.

We see some limitations in the present dataset. First, the sample size may be insufficient for the dataset to be considered as reference data for young and older participants. However, to our knowledge, this is the largest dataset to be made publicly available that includes diverse types of biomechanics, age, walking-environment, and gait-speed data. Second, the subjects performed the overground trials in a 10-m walkway due to the dimension limitation of the laboratory. Therefore, the present results should be interpreted with cautious since the self-selected gait speeds might have been slightly underestimated, relative to longer distance trials, as demonstrated by Seethapathi & Srinivasan (2015). Lastly, five participants in the Older group walked while holding the treadmill’s handrails (these participants are identified in the file that contains the metadata information), and, although their biomechanical patterns do not seem to differ from those of the other participants, this fact should be considered when using the dataset.

## Conclusions

The present study created a public dataset containing raw and processed kinematics and kinetics data on both overground and treadmill walking trials at a range of gait speeds in both young and older healthy adults. This dataset may be used to enhance knowledge related to the influence of age, environment, and walking speed on gait biomechanics. In addition, it may serve educational needs and, with the inclusion of additional participants, as normative gait data.

##  Supplemental Information

10.7717/peerj.4640/supp-1 Supplemental Information 1 Description of anatomical landmarks and ensemble average biomechanical waveforms for the Older group

## Additional Information and Declarations

### Data Availability

The following information was supplied regarding data availability: Fukuchi, Claudiane; Fukuchi, Reginaldo; Duarte, Marcos (2018): A public data set of overground and treadmill walking kinematics and kinetics of healthy individuals. figshare. Fileset. 10.6084/m9.figshare.5722711.v2.
