# Supplementary material for: A public dataset of overground and treadmill walking kinematics and kinetics in healthy individuals
Source: PeerJ. 2018 Apr 24;6:e4640. doi: 10.7717/peerj.4640 (PMC5922232; doi:10.7717/peerj.4640)
Supplement: Supplemental Information 1 [file peerj-06-4640-s001.docx]

**Table 1.** Details of the 28 anatomical reflective markers used to determine the position and orientation of the body segments during walking trials.

| **#** | **Label** | **Name** | **Description** |
| --- | --- | --- | --- |
| 1 | R.ASIS | Right Anterior Superior Iliac Spine | Right anterior superior iliac spine |
| 2 | L.ASIS | Left Anterior Superior Iliac Spine | Left anterior superior iliac spine |
| 3 | R.PSIS | Right Posterior Iliac Spine | Right posterior superior iliac spine |
| 4 | L.PSIS | Left Posterior Iliac Spine | Left posterior superior iliac spine |
| 5 | R.Iliac.Crest | Right Iliac Crest | Uppermost margin of the right iliac crest |
| 6 | L.Iliac.Crest | Left Iliac Crest | Uppermost margin of the left iliac crest |
| 7 | R.Heel.Bottom | Right Heel Bottom | Aspect of the Achilles tendon insertion on the right calcaneous |
| 8 | L.Heel.Bottom | Left Heel Bottom | Aspect of the Achilles tendon insertion on the left calcaneous |
| 9 | R.GTR | Right Greater Trochanter | Most lateral prominence of the right greater trochanter |
| 10 | R.Knee | Right Knee | Most lateral prominence of the right lateral femoral epicondyle |
| 11 | R.Knee.Medial | Right Knee Medial | Most medial prominence of the right lateral femoral epicondyle |
| 12 | R.HF | Right Head of Fibula | Proximal tip of the head of the right fibula |
| 13 | R.TT | Right Tibial Tuberosity | Most anterior border of the right tibial tuberosity |
| 14 | R.Ankle | Right Ankle | Lateral prominence of the right lateral malleolus |
| 15 | R.Ankle.Medial | Right Ankle Medial | Most medial prominence of the right medial malleolus |
| 16 | R.MT1 | Right 1^st^ Metatarsal | Dorsal margin of the right 1^st^ metatarsal head |
| 17 | R.MT5 | Right 5^th^ Metatarsal | Dorsal margin of the right 5^th^ metatarsal head |
| 18 | R.MT2 | Right 2^nd^ Metatarsal | Dorsal margin of the right 2^nd^ metatarsal head |
| 19 | L.GTR | Left Greater Trochanter | Most lateral prominence of the left greater trochanter |
| 20 | L.Knee | Left Knee | Most lateral prominence of the left lateral femoral epicondyle |
| 21 | L.Knee.Medial | Left Knee Medial | Most medial prominence of the left lateral femoral epicondyle |
| 22 | L.HF | Left Head of Fibula | Proximal tip of the head of the left fibula |
| 23 | L.TT | Left Tibial Tuberosity | Most anterior border of the left tibial tuberosity |
| 24 | L.Ankle | Left Ankle | Lateral prominence of the left lateral malleolus |
| 25 | L.Ankle.Medial | Left Ankle Medial | Most medial prominence of the left medial malleolus |
| 26 | L.MT1 | Left 1^st^ Metatarsal | Dorsal margin of the left 1^st^ metatarsal head |
| 27 | L.MT5 | Left 5^th^ Metatarsal | Dorsal margin of the left 5^th^ metatarsal head |
| 28 | L.MT2 | Left 2^nd^ Metatarsal | Dorsal margin of the left 2^nd^ metatarsal head |


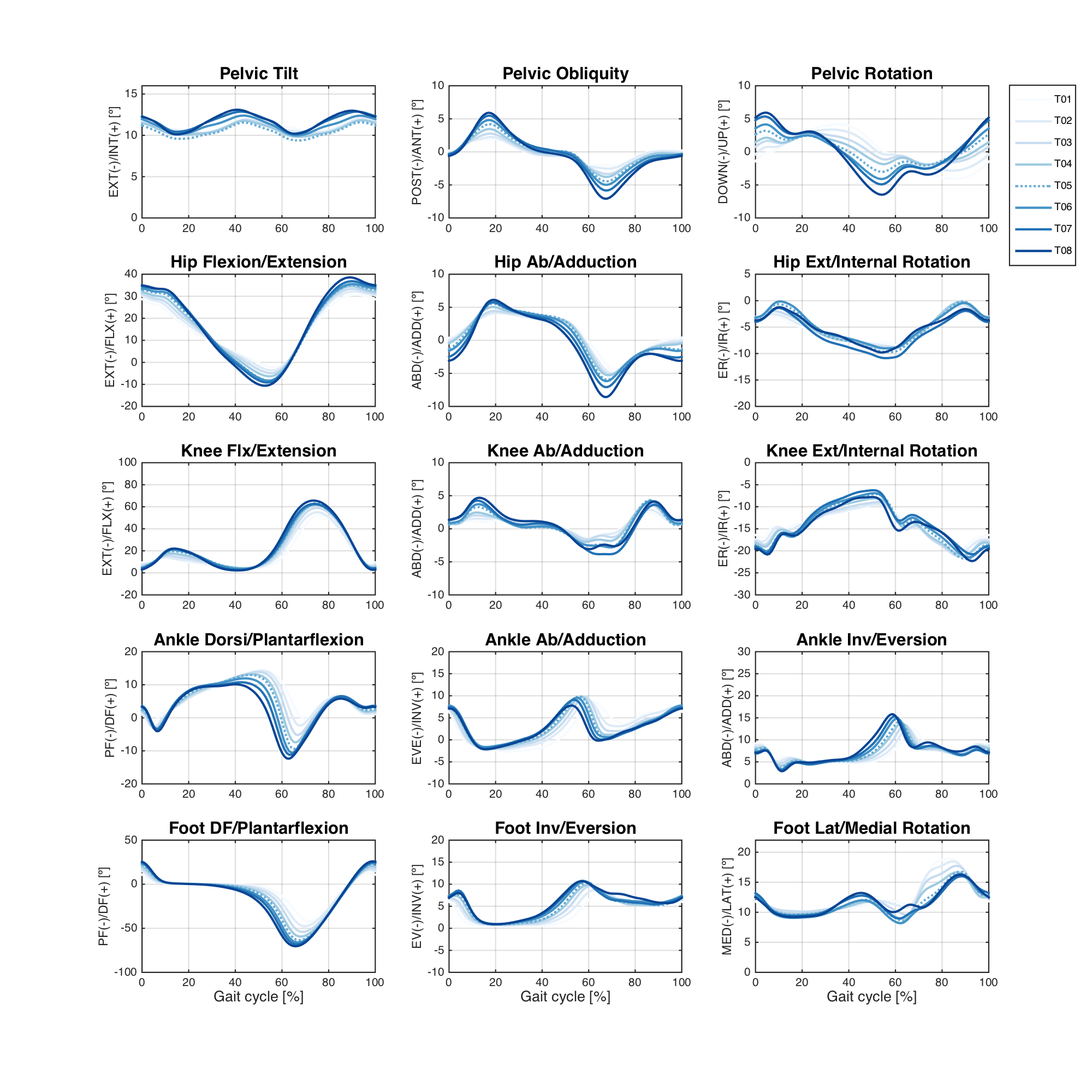


**Figure 1.** Ensemble average across Older group participants of the pelvis, hip, knee, ankle, and foot angles during the treadmill walking condition. Each waveform represents a walking speed (see legend).

*
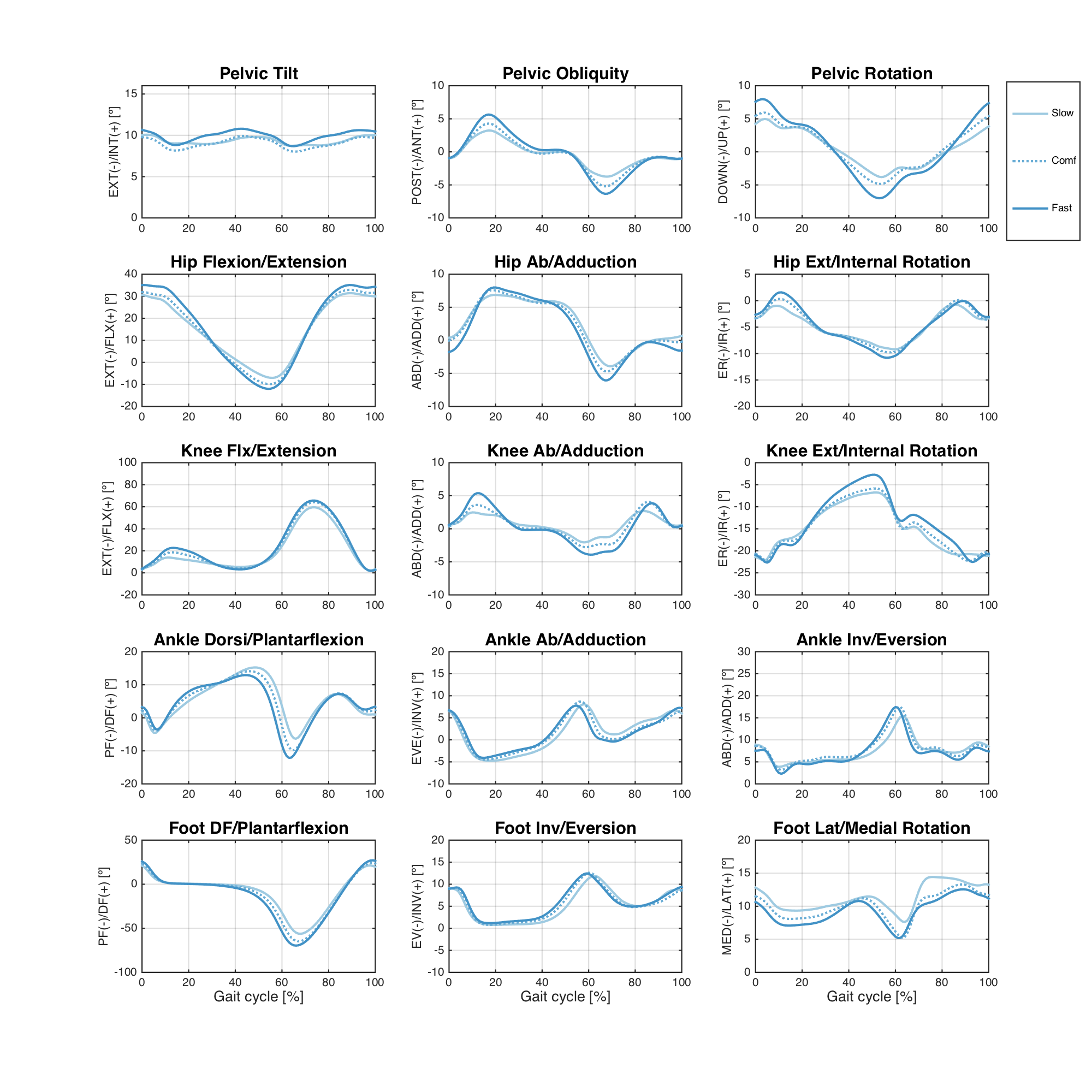
*

**Figure 2.** Ensemble average across Older group participants of the pelvis, hip, knee, ankle, and foot angles during the overground walking condition. Each waveform represents a walking speed (see legend).

*
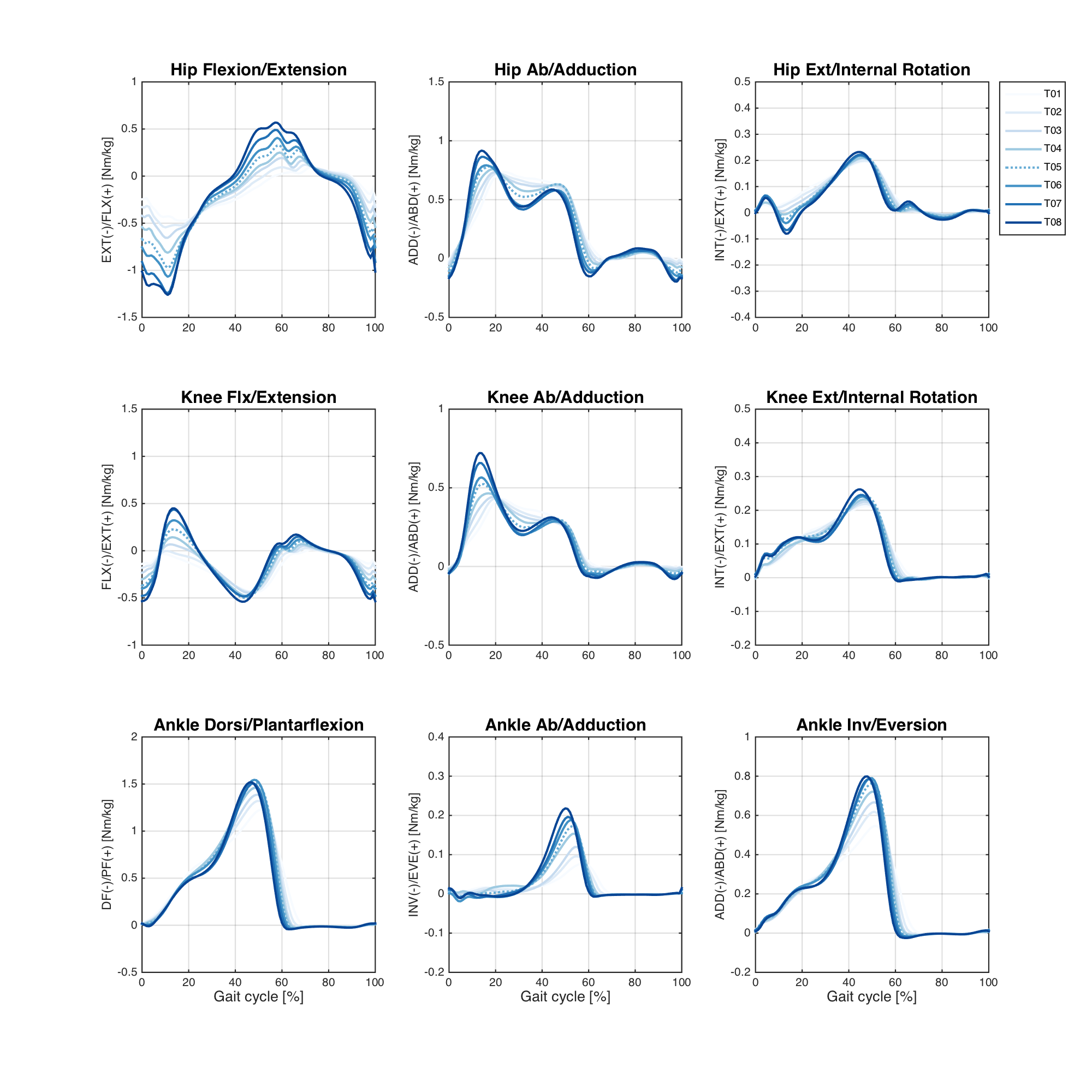
*

**Figure 3.** Ensemble average across Older group participants of the hip, knee, and ankle joint moments during the treadmill walking condition. Each waveform represents a walking speed (see legend).

*
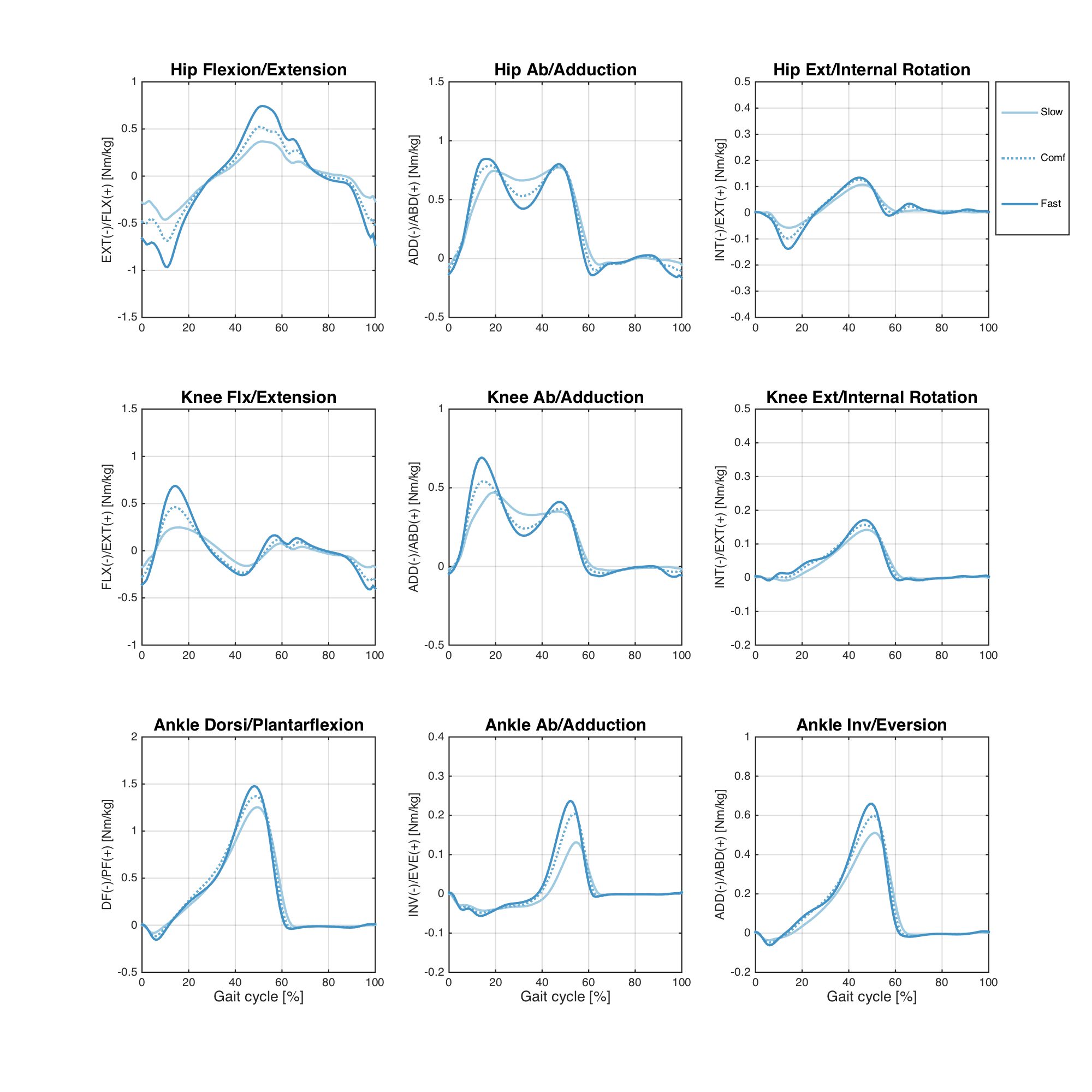
*

**Figure 4.** Ensemble average across Older group participants of the hip, knee, and ankle joint moments during the overground walking condition. Each waveform represents a walking speed (see legend).


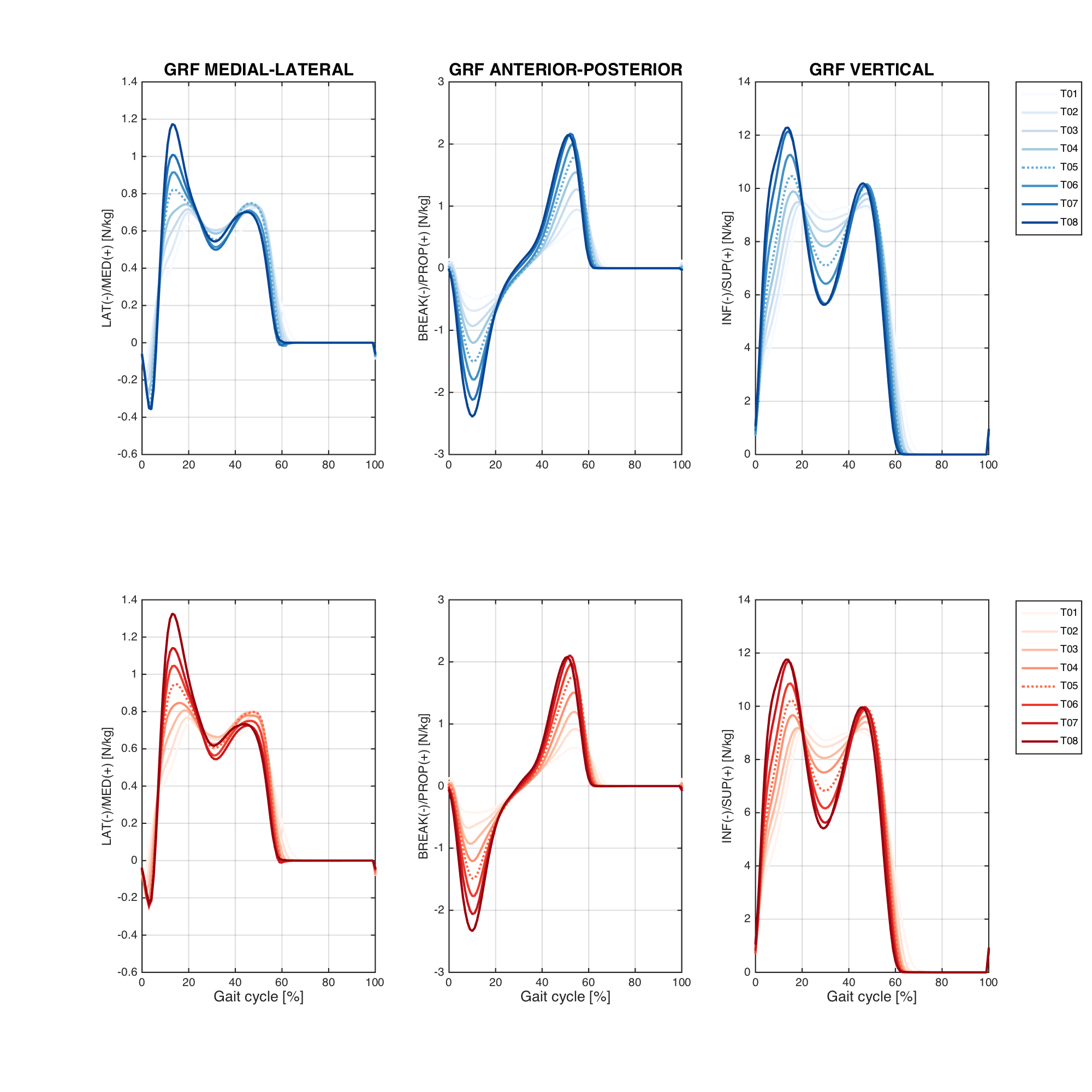


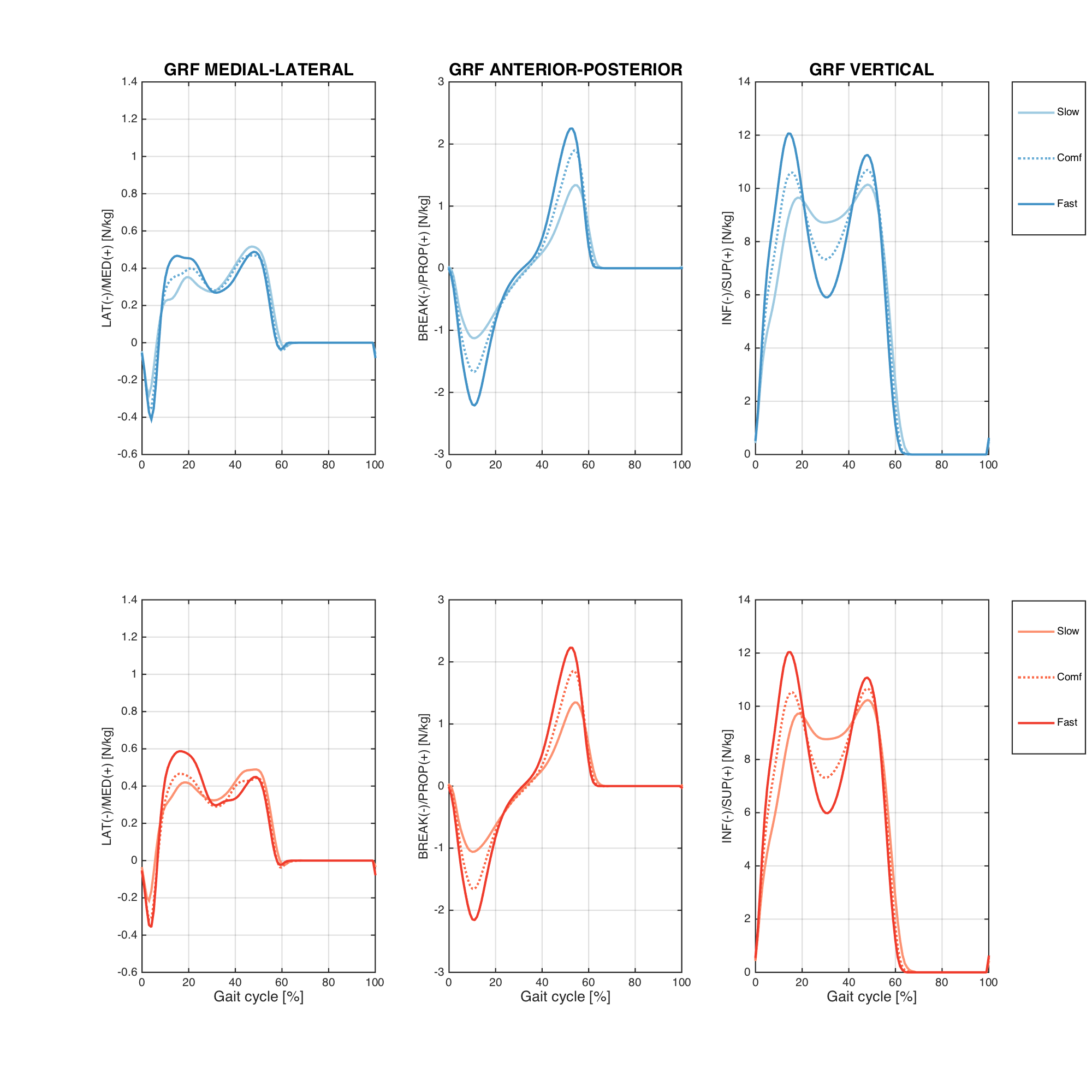


**Figure 5.** Ensemble average across Older group participants of the ground reaction force (GRF) on the treadmill (top) and overground (bottom) walking conditions. Each waveform represents a walking speed (see legend).
